# Beyond Access Block: Understanding the Role of Health Literacy and Self-Efficacy in Low-Acuity Emergency Department Patients

**DOI:** 10.31486/toj.19.0047

**Published:** 2020

**Authors:** Andrew Wayment, Curtis Wong, Sean Byers, Rob Eley, Mary Boyde, Remo Ostini

**Affiliations:** ^1^ The University of Queensland, Faculty of Medicine, Ochsner Clinical School, New Orleans, LA; ^2^ Emergency Department, Princess Alexandra Hospital, Woolloongabba, Queensland, Australia; ^3^ Faculty of Medicine, The University of Queensland, Brisbane, Australia; ^4^ Princess Alexandra Hospital, Brisbane, Queensland, Australia; ^5^ Rural Clinical School, The University of Queensland Faculty of Medicine, Toowoomba, Queensland, Australia

**Keywords:** *Emergency service–hospital*, *health care surveys*, *health literacy*, *self-efficacy*, *self-evaluation*

## Abstract

**Background:** Health literacy, self-efficacy, and patient satisfaction are factors associated with healthcare utilization. The relationships among these factors and their combined impact on patients’ self-rated health have historically been studied in chronic disease populations. This study assessed low-acuity emergency department (ED) patients’ ratings of these factors, the relationships among these factors, and their effect on re-presentation rates to the ED.

**Methods:** In this single-arm cohort survey, patients provided demographic data, completed health literacy and self-efficacy assessments prior to being seen by a physician, and completed a discharge perceptions questionnaire that included a global satisfaction question at the time of departure. Three months later, patients answered a telephone survey to measure post-ED visit health outcomes.

**Results:** Health literacy (*r*=0.114, *P*=0.023) and self-efficacy (*r*=0.469, *P*<0.001) were both independently and positively associated with self-rated health. Neither factor was associated with patient satisfaction. Self-rated health was negatively associated with return ED visits (*r*=–0.137, *P*=0.011).

**Conclusion:** Existing research shows that health literacy has a linear association with self-efficacy and self-rated health. The results of this study suggest that in the context of low-acuity ED patients, health literacy and self-efficacy affect patients’ understanding of their health status (self-rated health) but do not lead to better utilization of healthcare resources. Improvement of health literacy and self-efficacy, specifically to increase self-rated health, may provide a future avenue of intervention to reduce low-acuity ED patient re-presentation.

## INTRODUCTION

In 2015-2016, emergency department (ED) visits across Australia rose to approximately 7.5 million presentations from 6.5 million in 2011-2012, representing a 3.8% average increase in ED presentations per year during that time frame.^1^ Concurrently, national ED expenditures increased from $3.4 billion to $4.7 billion, an increase of 38.2%,^2,3^ with the average cost of ED presentations increasing by 13.4% from $575 to $652 per patient.^2,4^ ED presentations continued to rise across all Australian states and territories in 2016-2017, climbing to nearly 7.8 million presentations nationwide.^5^ The upward trend of ED presentations in Australia reflects similar trends in the United States, Canada, and the United Kingdom.^6-9^

Access block, or boarding, which is the delay in patient admission from the ED because of a lack of inpatient bed capacity, is often cited in the literature as being the ultimate driver of ED overcrowding and strain.^10,11^ However, rising numbers of nonadmitted ED patients are increasingly adding to the overall ED burden. In fact, the overall increase in the average cost of ED presentations has been reported to be driven entirely by an increase in the average cost per nonadmitted ED presentation. Between the 2011-2012 and 2015-2016 financial years, the cost of nonadmitted ED presentations increased 16.7%, from $443 to $517 per patient; conversely, the average cost per admitted ED presentation decreased 6.5%, from $1,045 to $977 per patient.^2-4^ Currently, nonadmitted expenditures constitute 56% of total ED expenditures.^2^ While a large driver of costs can be attributed to system-level phenomena such as increased frequency of diagnostic testing,^12-15^ the contribution of patient-level factors among different patient subgroups warrants investigation.

One subgroup of interest among nonadmitted ED patients is low-acuity ED patients. The Australian Institute of Health and Welfare refers to these patients as general practitioner (GP)–type presentations whose ED visits could have been avoided through the provision of nonhospital health services. Low-acuity ED patients are allocated a triage score indicating lower urgency to be seen upon presentation to the ED, do not arrive by ambulance, are not admitted to the hospital, and do not die.^16^

Among the numerous reasons for this type of ED presentation are GP unavailability, referral from a patient's GP, excess waiting time for a GP, inadequate GP equipment or facilities, and, simply, patient preference.^5,17^ Previous studies attempting to describe or characterize this population and the factors driving their healthcare utilization have often focused on demographic characteristics: ethnicity, socioeconomic status, mental health status, marriage status, and employment.^18-21^ Other factors associated with healthcare utilization are health literacy,^22,23^ perceived self-efficacy, and patient satisfaction.^24^ Unlike demographics, these factors are more modifiable and play broad roles in both patient healthcare utilization and the management of chronic conditions.

Health literacy is the set of patients’ cognitive and social skills that allows them to access, understand, and use information in ways that promote and maintain their health.^25^ Low health literacy has long been associated with increased ED usage.^22,26-30^ Additionally, lower health literacy has been shown to be associated with nonadherence to care instructions, higher rates of re-presentation, poor health outcomes,^23^ and lower levels of self-reported health.^31,32^

Self-efficacy is the perception of one's own ability to implement behaviors to attain an outcome.^33^ Previous research has found associations between patient health literacy and self-efficacy, resulting in the theory that patients with higher health literacy will better understand their conditions and thus feel that they have a greater ability to manage their own care.^34-39^ However, such research has focused on specific chronic disease patient populations, such as patients with type 2 diabetes mellitus; these factors have not been examined in low-acuity ED patients. Self-efficacy, independent of other factors, is positively associated with self-care adherence and self-management.^34,40-42^ These studies showed that patients who felt that they had better ability to manage their own condition were more likely to carry out self-management. Through better self-management, patients with higher self-efficacy may present to the ED less often.

Patient satisfaction is a highly subjective measure of the patient experience, with no clear consensus on definition.^43^ However, patient satisfaction has been found to have an impact on quality improvement of care and has demonstrated an association with patient health literacy.^23,43^ While the nature of the patient satisfaction and health literacy relationship remains unclear, both have been associated with poor health outcomes.^27,28,44^ Patient satisfaction can be considered an inherent part of a patient's unique profile and influences patient perspectives, decision making, and self-management.

Given the increasing patient load on emergency medical services, the relative lack of information on low-acuity ED patients, and the potential opportunity for patient health literacy and self-efficacy to provide a point of intervention, the relationship between these factors is an area that requires investigation. The primary objective of this study was to determine low-acuity ED patients’ ratings of health literacy, self-efficacy, patient satisfaction, and self-rated health and to assess the relationships among these factors. The secondary objective of this study was to identify how these factors were associated with patient triage categorization and patient re-presentation to the ED.

## METHODS

### Study Design and Setting

The study used a single-arm cohort survey with follow-up of low-acuity ED adult patients in a large tertiary hospital located in a major metropolitan area. The study had 3 phases. In phase 1, patients provided demographic data and completed health literacy and self-efficacy assessments prior to being seen by a physician. At discharge, they completed a discharge perceptions questionnaire. In phase 2, approximately 3 months following discharge, patients received a follow-up telephone call and answered questions from a researcher-administered short survey regarding their postdischarge health and whether they returned to the ED. In phase 3, patient triage scores, admission status, re-presentation status, and length of stay in the month following the initial ED visit were obtained from patient health records to confirm patient acuity, verify eligibility, and investigate re-presentation. The study was approved and monitored by the Metro South Health Human Research Ethics Committee.

### Selection of Participants

Study participants were limited to adult patients who ambulated into the Princess Alexandra Hospital (Woolloongabba, Queensland, Australia) ED waiting room, were not immediately admitted to acute emergency care, and had an Australasian Triage Scale (ATS) score of 3 to 5. The ATS is a triage scale similar to the Emergency Severity Index (ESI) used in the United States.^45,46^

Exclusion criteria included any patient who was brought to the hospital by ambulance, patients with observable altered mental status or physical aggression, non-English speakers, patients <18 years, and any patients who were subsequently admitted to acute emergency care or to any other hospital unit during their visit.

**Figure 1.**
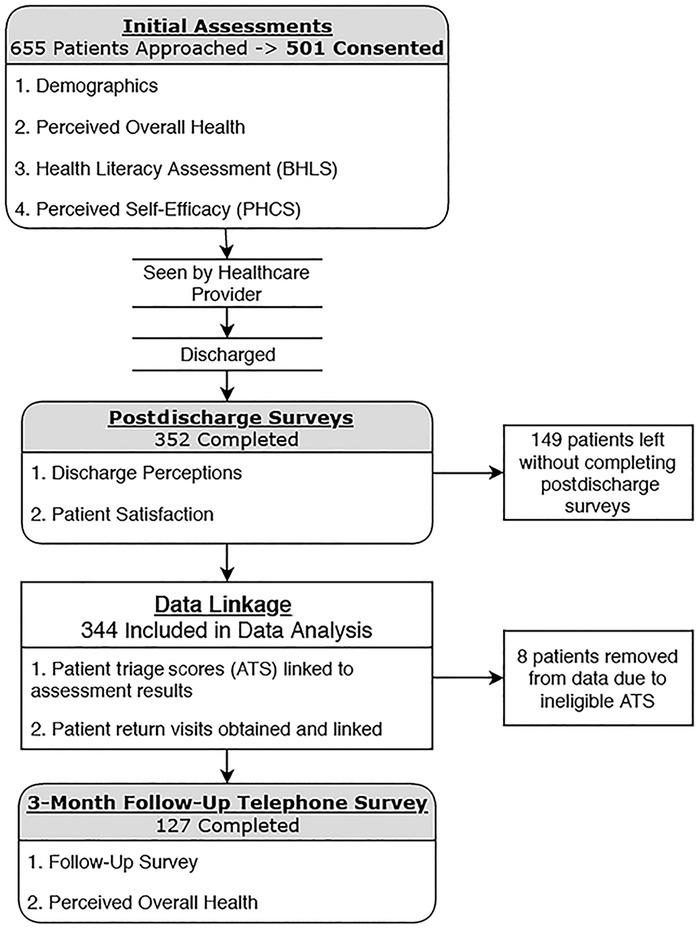
**Diagram of study method and study sample numbers.** ATS, Australasian Triage Scale; BHLS, Brief Health Literacy Screen; PHCS, Perceived Health Competence Scale (Perceived Medical Condition Self-Management Scale).

Trained researchers approached 655 patients in the ED waiting room from 8:00 am to 10:00 pm each day for a period of 4 weeks, starting in November 2017. Recruiters were counseled to not approach any patient exhibiting aggression or altered mental status. Any question regarding a patient's eligibility for the study was deferred to the ED nurse in triage. Eight patients were excluded from data analysis because they were discovered to have a triage score of 1 or 2 or because they were accidentally recruited after being admitted and thus did not meet the inclusion criteria. A total of 344 patients completed the study and were included in the analysis (Figure 1).

### Measurements

### Phase 1

#### Personal Characteristics and Self-Rated Health

Patient data regarding age, sex, level of education, and primary language were collected. Additionally, patients rated their overall health on a 5-point Likert scale—ranging from very good to very poor—used by the World Health Organization. This score was used as a measure of the patient's self-rated health.^47^

#### Health Literacy

**Figure 2.**
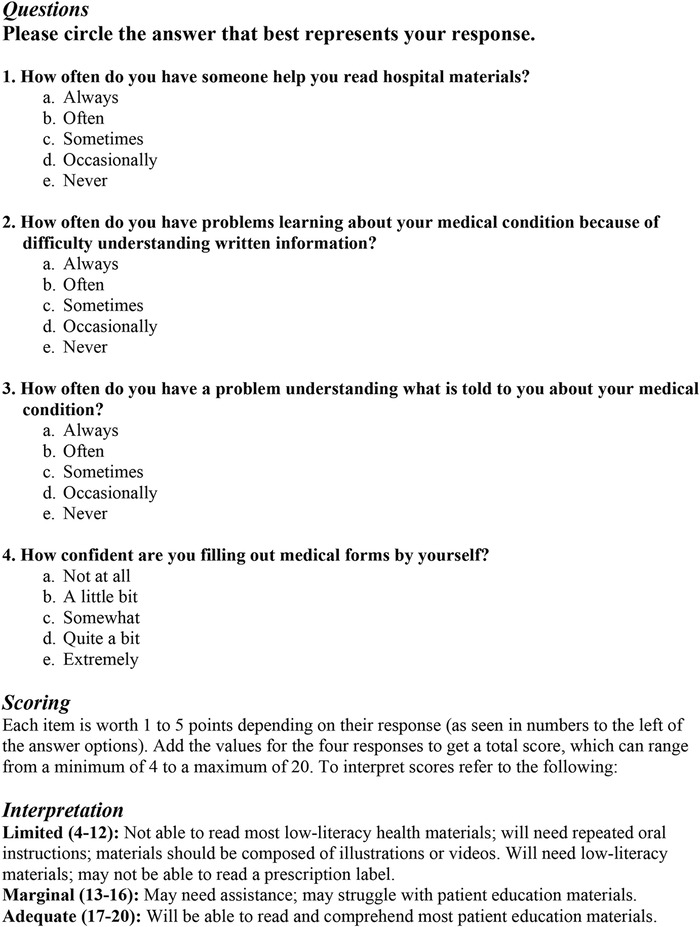
**Brief Health Literacy Screen: questions, scoring, and interpretation.**

Health literacy was assessed using the Brief Health Literacy Screen (BHLS),^48^ a 4-question assessment that patients completed on tablets (Figure 2). Visually impaired patients answered the questionnaire with the help of a researcher who read the questions aloud. Each question was worth 1 to 5 points based on the patient's response, and points were summed to give a score ranging from 4 to 20. Patients scoring lower on the BHLS struggle with reading patient education materials and prescription labels and may require oral instructions or low-literacy reading materials. Patients who score adequately (ie, 17 to 20 points) can read and comprehend most patient education materials. The BHLS has been validated in multiple studies^48-53^ conducted in several settings^54,55^ and was selected because of its ease of administration and scoring relative to other health literacy assessments, such as the Short Test of Functional Health Literacy in Adults. Ease of administration and ease of scoring are important attributes, particularly given how quickly patients can move through the ED.^48^

#### Perceived Self-Efficacy

**Figure 3.**
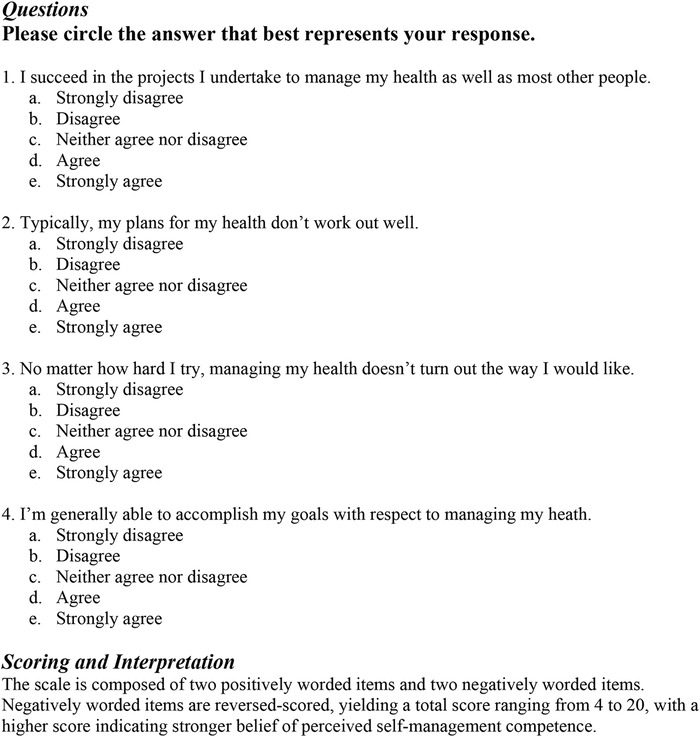
**Shortened Perceived Medical Condition Self-Management Scale: questions, scoring, and interpretation.**

Perceived self-efficacy was measured using a validated short-form, 4-question version of the Perceived Medical Condition Self-Management Scale (Figure 3).^56-58^ Each question was scored using a 5-point Likert scale, and points were summed to give a total score ranging from 4 to 20. A higher score indicates stronger belief of perceived self-management competence. As with the BHLS, patients completed the assessment on tablets, with visually impaired patients answering the questionnaire with the help of a researcher.

#### Discharge Perceptions and Patient Satisfaction

Patient discharge is a highly coordinated group effort involving ED personnel. Doctors, nurses, and support staff work with patients to provide treatment, educate patients about their complaint and necessary management, and arrange appropriate follow-up actions. The patient is the sole connection between an ED visit and follow-up management, so discharge is a vulnerable and critical time point in ED patient care and hinges on the patient's ability to understand the diagnosis, treatment, and management of the condition, as well as the patient's ability to successfully implement a care plan.^59^ Given this significance, a modified patient hospital experience questionnaire was used to assess patient discharge perceptions to characterize some of the effects that health literacy and perceived self-efficacy might have on the discharge process. Validated discharge perception questions were taken from the Queensland Health Emergency Department Patient Experience Survey 2013 and modified for use in this study.^60,61^ Following discharge, patients answered 9 yes/no questions regarding their healthcare usage (eg, whether they had a regular doctor); their perception of their presenting complaint (eg, whether they felt a GP could have assessed their complaint); and their understanding of the diagnosis, treatment, and follow-up. An additional yes/no question, which was used to gauge patient satisfaction, asked patients if they were satisfied with their experience in the ED.

### Phase 2

#### Follow-Up Phone Call

Approximately 3 months after discharge, the medical student authors made follow-up telephone calls to patients. A maximum of 3 attempts were made to reach patients. In total, 127 patients (36.9%) completed the follow-up phone call, answering a 5-question survey regarding their health following discharge and whether they required additional care for their presenting complaint. Patients were also asked the same self-rated health question—responses ranged from very good to very poor—that they had answered during the initial assessment in the ED.

### Phase 3

#### Data Linkage

Patient triage scores, admission status and length of stay, and re-presentation status in the month following their visit were obtained from patient health records. This information was also used to identify patients who did not meet inclusion criteria in terms of admission or triage category.

### Statistical Analysis

Descriptive statistics including means ± standard deviations for continuous variables and proportions for categorical variables were calculated for participant demographic variables and triage scores. Bivariate correlations were determined using Spearman correlations for all nonparametric variables, including self-rated health, health literacy, and self-efficacy. Independent relationships with health literacy and self-efficacy as predictors and self-rated health as the outcome were modeled using multiple linear regression with age, education, and primary language as covariates. Statistical significance for this study was defined as *P*<0.05. All statistical analysis was undertaken using SPSS v.18.0 (IBM Corp.).^62^

## RESULTS

### Characteristics of Study Subjects

Table 1 presents the demographic characteristics of the study sample. Average participant age was 40.29 ± 16.1 years; 131 (38.1%) patients were female, 209 (60.8%) were male, and 4 (1.2%) identified as other. Table 2 shows the statistical associations between study participant demographic characteristics and triage scores with the assessed psychosocial factors, length of stay, and patient satisfaction. A number of factors (health literacy, self-efficacy, and length of stay) were associated with education, age, primary language, and triage score. No measured factors were associated with sex or patient satisfaction.

**Table 1.** Descriptive Characteristics of Participants

Variable	All Patients (n=344)
Age, years, mean ± SD	40.29 ± 16.1
Sex	
Female	131 (38.1)
Male	209 (60.8)
Other	4 (1.2)
Primary language	
English	303 (88.1)
Non-English	41 (11.9)
Education	
School level	104 (30.2)
Certificate level	65 (18.9)
Advanced diploma/diploma level	31 (9.0)
Bachelor degree level	90 (26.2)
Graduate diploma/certificate level	23 (6.7)
Postgraduate degree level	31 (9.0)
Australasian Triage Scale score	
3	136 (39.5)
4	155 (45.1)
5	53 (15.4)
Brief Health Literacy Screen score, mean ± SD	17.46 ± 2.9
Perceived Medical Condition Self-Management Scale score, mean ± SD	14.83 ± 2.94
Self-rated overall health	
Very poor	4 (1.2)
Poor	12 (3.5)
Moderate	59 (17.2)
Good	176 (51.2)
Very good	93 (27.0)

Note: Data are presented as n (%) unless otherwise indicated.

**Table 2.** Study Sample Demographics and Associations With Health Literacy, Perceived Self-Efficacy, Self-Rated Health, Length of Stay, and Satisfaction

Variable	Health Literacy, r_s_ (*P* Value)	Perceived Self-Efficacy, r_s_ (*P* Value)	Self-Rated Health, r_s_ (*P* Value)	Length of Stay, r_s_ (*P* Value)	Satisfaction, r_s_ (*P* Value)
Age	0.088 (>0.05)	0.115 (0.033)	0.031 (>0.05)	0.109 (0.044)	0.020 (>0.05)
Sex	–0.015 (>0.05)	–0.092 (>0.05)	–0.061 (>0.05)	0.055 (>0.05)	–0.038 (>0.05)
Education	0.128 (0.018)	0.053 (>0.05)	0.155 (0.004)	–0.106 (0.050)	–0.096 (0.05)
Language	0.265 (<0.001)	0.122 (0.024)	0.002 (>0.05)	–0.003 (>0.05)	0.035 (>0.05)
Australasian Triage Scale score	–0.087 (>0.05)	–0.015 (>0.05)	0.017 (>0.05)	–0.151 (0.005)	0.018 (>0.05)

r_s_, Spearman rank order correlation.

### Ratings, Relationships, and Re-Presentation

The mean health literacy score using the BHLS was 17.46 ± 2.9 of a possible score of 20 (Table 1), reflecting that 70.6% of study participants had adequate overall health literacy. The health literacy score of patients with an ATS score of 5 (16.75 ± 3.3) was lower than that of patients with an ATS score of 3 (17.73 ± 2.7) (*P*=0.037).

As shown in Table 3, health literacy was positively associated with self-rated health at initial assessment and with self-efficacy. Health literacy was not significantly correlated with patient satisfaction. At both the initial and follow-up assessments, self-efficacy had a stronger association with self-rated health than health literacy but was not associated with patient satisfaction.

**Table 3.** Associations Between Health Literacy, Perceived Self-Efficacy, Self-Rated Health, Length of Stay, and Satisfaction

Factor	Health Literacy, r_s_ (*P* Value)	Perceived Self-Efficacy, r_s_ (*P* Value)
Perceived self-efficacy	0.399 (<0.001)	
Self-rated health at emergency department presentation	0.329 (<0.001)	0.518 (<0.001)
Self-rated health at follow-up	0.172 (>0.05)	0.305 (0.001)
Length of stay	–0.044 (>0.05)	0.070 (>0.05)
Satisfaction	0.074 (>0.05)	0.087 (>0.05)

r_s_, Spearman rank order correlation.

In addition to health literacy and self-efficacy, initial self-rated health was positively correlated with self-rated health at follow-up (r_s_=0.224, *P*=0.012). Modeled results show that health literacy (*r*=0.114, *P*=0.023) and self-efficacy (*r*=0.469, *P*<0.001) have significant independent effects on self-rated health, even when controlling for variables such as age, education, and primary language (overall model R-squared=0.285, F(5, 338)=26.93, *P*<0.001) (data not shown).

Self-rated health was negatively correlated with patients returning to the ED (*r_s_*=–0.137, *P*=0.011) and was the only significant predictor of re-presentation.

### Discharge Perceptions Results

Table 4 presents the summary of responses to the discharge perceptions survey and their associations with health literacy and self-efficacy. Question 1—which asked patients if they felt their visit was an emergency—had a weak association with health literacy and no relationship with self-efficacy. Slightly more than one-third of patients (34.9%) felt that their presenting complaint was *not* an emergency. Similarly, 32.0% of patients felt that their presenting complaint could have been treated at a GP office (question 3). Questions 1 and 3 had a weak negative association (*r_s_*=–0.165, *P*=0.002), meaning that the more likely a patient felt that the visit was an emergency, the less likely the patient felt that a GP could have treated the problem. Questions 5 and 6—which asked patients if they understood their diagnosis and treatment, respectively—also had no association with health literacy or self-efficacy. Ninety-three percent of patients felt that they understood their diagnosis, and 92.2% felt that they understood the treatment of their diagnosis.

**Table 4.** Discharge Perceptions Responses and Associations With Health Literacy and Perceived Self-Efficacy

Question	Yes, %	Health Literacy, r_s_ (*P* Value)	Perceived Self-Efficacy, r_s_ (*P* Value)
1. Was the reason you came here today an emergency?	65.1	–0.113 (0.036)	–0.100 (>0.05)
2. Have you been to the ER for the same reason before?	28.2	–0.115 (0.033)	–0.081 (>0.05)
3. Do you think your medical problem today could have been treated at a general practice, by a GP?	32.0	0.018 (>0.05)	–0.017 (>0.05)
4. Do you have a doctor that you see on a regular basis?	75.6	–0.013 (>0.05)	0.086 (>0.05)
5. Do you understand what your diagnosis was today?	93.0	0.103 (>0.05)	0.049 (>0.05)
6. Do you understand the treatment of your diagnosis?	92.2	0.084 (>0.05)	0.014 (>0.05)
7. Do you know what the next step is in the management of your diagnosis?	92.4	0.049 (>0.05)	0.059 (>0.05)
8. Do you have a follow-up appointment scheduled?	40.7	–0.085 (>0.05)	–0.043 (>0.05)
9. If you answered YES to the above question, are you likely to go to your follow-up appointment?	44.5	–0.057 (>0.05)	0.018 (>0.05)

ER, emergency room; GP, general practitioner; r_s_, Spearman rank order correlation.

## DISCUSSION

To our knowledge, this study is the first to measure health literacy, self-efficacy, and patient satisfaction in low-acuity ED patients and the first study to attempt to assess the connections between these factors and health outcomes such as health status and re-presentation rates.

**Figure 4.**
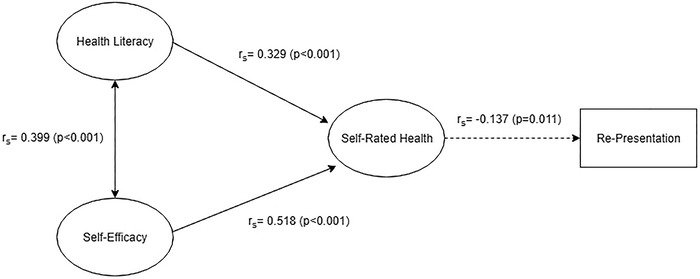
**Descriptive model based on study results.**

This study found a positive association between health literacy and self-efficacy. Health literacy and self-efficacy also had independent positive associations with self-rated health, and self-rated health had a negative association with re-presentation. The latter association suggests that sicker patients are more likely to return to the ED, which is entirely appropriate. These findings suggest a triangular model (Figure 4) in which health literacy and self-efficacy work synergistically to increase perceived self-rated health.

This model differs from the pathways modeled in previous literature studying the connections between health literacy, self-efficacy, and self-care behaviors in patients with type 2 diabetes mellitus.^34,37,63^ As with this study, previous studies found that health literacy affected self-efficacy, which itself was correlated with increased self-care behaviors such as hemoglobin A1c control. However, these previous studies found no significant association between health literacy and self-care behaviors, suggesting a sequential, linear relationship between health literacy, self-efficacy, and self-care behaviors.^34,37,63^ Our study found independent positive associations between both health literacy and self-efficacy, with self-rated health and self-efficacy having a stronger relationship.

We did not find any significant associations between patient satisfaction and any other measures we examined, including health outcomes (eg, self-rated health). Because of the paucity of published research on the topic, we cannot determine if our findings differ from previous research in this regard.

The results of the discharge questionnaire showed a weak negative association between whether patients felt that their visit was an emergency and whether they could have been treated by a GP, indicating that the more patients felt that their visit was an emergency, the less likely they felt that a GP could have treated the problem. This finding further implies that a subset of patients was aware that they were visiting the ED with a problem that could have been treated at a GP office.

Additionally, health literacy and self-efficacy had weak positive and negative associations, respectively, with whether patients felt their visit was an emergency. The discharge questionnaire results showed no association between health literacy, self-efficacy, and whether patients felt that they understood their diagnosis and treatment, a surprising result given how health literacy, by definition, underscores a person's ability to understand his/her own health.

These findings collectively suggest that health literacy and self-efficacy work in this population by enhancing patients’ understanding of how healthy they are (ie, self-rated health) instead of leading to better utilization of healthcare resources, which is demonstrated by the pivotal role self-rated health plays in this study's model and the weak statistical association of health literacy and self-efficacy with patients’ decisions to present to the ED.

Consequently, changing current low-acuity ED visit trends may be achieved by targeting self-rated health or the perception of self-rated health. Cheng et al evaluated the use of empowerment sessions to improve empowerment perceptions and indirectly improve self-management.^64^ In a randomized controlled trial of patients with type 2 diabetes mellitus, they found significant improvement in the intervention group that received empowerment sessions. Lee et al demonstrated that empowerment perceptions directly affect health literacy.^34^ If this specific relationship also holds true in the low-acuity ED patient population, empowerment sessions may be a means of changing health literacy, self-rated health, and, ultimately, ED visit trends. The efficacy of providing empowerment sessions to low-acuity ED patients is a potential direction for future research.

### Limitations

This study has 5 primary limitations. First, this was a single-center study located in a metropolitan area in a developed country. How these findings might translate to rural areas of Australia or less-developed countries worldwide is unknown. Second, a substantial number of patients (n=149) consented and completed initial assessments but did not complete discharge assessments; whether this group of patients is similar to the final study sample is unknown. Because of our data collection process, the demographic information for these patients was not retained, preventing us from comparing the 2 groups. Thus, the study population in this study could be very specific and thus not generalizable to the greater low-acuity ED population. Third, the timing of the discharge questionnaire administration may have been a limitation. After sitting in the waiting room and being evaluated by a physician, patients eager to leave may have completed the discharge questionnaire without giving it their full attention. Alternatively, patients may have understood verbal discussions and instructions with their care providers during their ED visit, but they may not have had the opportunity to read the discharge literature and written instructions. Patient understanding of these materials is highly dependent upon health literacy.^65,66^ The very high proportion of positive responses to the discharge questions means that a ceiling effect may have limited the ability of the statistical analysis to detect an effect. Fourth, we relied solely on patients’ self-reports, making subject response bias a limitation of this study. Fifth, the discharge questionnaire created for this study modified validated questions that have been used for years in national ED patient surveys in the United Kingdom and Australia. This modification was necessary because validated questions specifically pertaining to some of the topics explored in this study did not exist. Efforts were made to mirror the structure of previously validated questions. While the questions used in the discharge questionnaire are not validated, the results of the discharge questionnaire played no role in the study's primary and secondary objectives and were used to elucidate other aspects of the low-acuity ED population.

## CONCLUSION

Health literacy and self-efficacy were both independently and positively associated with self-rated health, with self-efficacy having the stronger effect. Health literacy and self-efficacy were positively associated with each other. Patient satisfaction was not related to health literacy, self-efficacy, self-rated health, or patient re-presentation. Self-rated health had a significant negative association with patient re-presentation to the ED among low-acuity patients.

## Notes

This article meets the Accreditation Council for Graduate Medical Education and the American Board of Medical Specialties Maintenance of Certification competencies for Patient Care, Medical Knowledge, and Systems-Based Practice.
